# Hexaploid-bridged introgression broadens genetic diversity and enriches clubroot-resistance alleles in *Brassica juncea*

**DOI:** 10.3389/fpls.2025.1729594

**Published:** 2026-01-22

**Authors:** Yongzhi Wu, Jingyan Liao, Yinquan Deng, Fuquan Ce, Peng Bai, Lisha Peng, Xingxing Zhang, Yi Wan, Wei Qian, Jiaqin Mei

**Affiliations:** 1 Integrative Science Center of Germplasm Creation in Western China (CHONGQING) Science City, Chongqing Technology Innovation Center of Breeding, Southwest University, Chongqing, China; 2 Academy of Agricultural Sciences, Southwest University, Chongqing, China; 3 State Cultivation Base of Crop Stress Biology for Southern Mountainous Land of Southwest University, Chongqing, China; 4 Changshou District Agricultural Technology and Scientific Research Service Center, Chongqing, China; 5 Southeast Chongqing Academy of Agricultural Sciences, Chongqing, China; 6 Fuling Academy of Southwest University, Chongqing, China; 7 Shudu Middle School, Chongqing, China

**Keywords:** clubroot resistance, genetic introgression, hexaploid bridge, marker-assisted selection, new-type Brassica juncea

## Abstract

**Introduction:**

The narrow genetic base of current *Brassica juncea* (AABB) has limited the genetic improvement of this crop.

**Methods:**

In this study, we developed novel germplasm by crossing synthetic hexaploid bridge lines (AABBBB and AAAABB) with diploid progenitors *B. rapa* (AA) and *B. nigra* (BB), followed by successive selfing and marker-assisted selection.

**Results and Discussion:**

Although the F1 hybrids exhibited wide variation in fertility and morphology, these traits stabilized at levels comparable to current *B. juncea* by the F2 and F3 generations. Whole-genome resequencing confirmed that the new-type lines not only constitute a unique genetic group but also show a genomic shift toward the current *B. juncea* cluster in the F2 populations. Consequently, these new-type lines represent a significant new source of genetic diversity. As a practical application, we introgressed clubroot resistance from the European fodder turnip ECD04 (*B. rapa*) into *B. juncea* using the hexaploid bridge strategy coupled with MAS. Over four selection cycles (F2 to F5), the donor allele frequency surged from 31.3% to 54.4%, showing a strong negative correlation with disease severity (r = -0.98) and yielding highly resistant F5 lines.

**Conclusions:**

This study demonstrates that the hexaploid bridge strategy enables rapid genetic base broadening and efficient trait improvement in *B. juncea*.

## 1 Introduction

*Brassica juncea* (*B. juncea*, AABB, 2n = 36), an amphidiploid species originating from natural hybridization between the diploid progenitors *Brassica nigra* (*B. nigra*, BB, 2n = 16) and *Brassica rapa* (*B. rapa*, AA, 2n = 20) and spontaneous chromosome doubling (Kang et al., 2021). It is widely cultivated as a multipurpose crop for oilseed, vegetable, and condiment production (Tian and Deng, 2020). This species is valued for its high adaptability to abiotic stresses, including drought (Chen et al., 2013b), salinity, and pod-shattering resistance (Rahman et al., 2022). These attributes make it well-suited for cultivation in marginal environments (Limbalkar et al., 2021). However, compared to its diploid progenitors, the genetic base of cultivated *B. juncea* remains narrow (Hittorf et al., 2020; Zhang et al., 2022), limiting its potential for further genetic improvement through conventional breeding (Singh et al., 2022).

Traditional approaches for gene introgression from diploid progenitors into *B. juncea*, such as direct hybridization between *B. rapa* and *B. nigra*, are hindered by strong pre- and post-zygotic reproductive barriers, including hybrid sterility and embryo abortion, often necessitating embryo rescue with low success rates (Liu et al., 2018). Moreover, backcrossing resistance genes from *B. rapa* into *B. juncea* is labor-intensive and time-consuming, typically requiring six to eight generations to achieve sufficient recovery of the recipient genome (Zhu et al., 2022). To overcome these limitations, hexaploid bridge lines with the AABBBB or AAAABB genome, developed from crosses of *B. juncea* with *B. rapa* or *B. nigra*, have demonstrated enhanced crossability with the other diploid progenitor and exhibited stable fertility (> 90% pollen viability and 2–4 seeds per pod) from the S1 to S3 generation, enabling efficient introgression of alleles from diploid progenitors into *B. juncea* under field conditions (Mei et al., 2022).

Although the synthetic hexaploid bridge strategy has demonstrated potential for introducing exotic genomic segments into *B. juncea* (Mei et al., 2022), the fate of these segments under continuous selfing remains poorly understood, particularly regarding fertility restoration, phenotypic stabilization, and genomic composition across generations (Yang et al., 2022a). It is well known that early-generation interspecific hybrids often suffer from meiotic abnormalities, including multivalent formation and genome loss, leading to compromised fertility (Xiong et al., 2011; Gaeta et al., 2007). Whether fertility and phenotypic stability can be restored in subsequent generations is critical to the practical utility of this strategy.

In addition to broadening genetic variation, the hexaploid bridge also offers a foundation for developing novel germplasm with key agronomic traits such as resistance to clubroot disease (caused by *Plasmodiophora brassicae*), which is absent in the current *B. juncea* gene pool but present in *B. rapa*. European fodder turnip ECD04 is a well-known *B. rapa* line that carrying multiple CR loci and exhibits strong resistance to several *Plasmodiophora brassicae* races (Yang et al., 2022b). To systematically introgress these CR genes from ECD04 into the *B. juncea* background, marker-assisted selection (MAS) offers a viable approach for tracking and enriching desirable resistance alleles in early generations (Tomita et al., 2013; Dakouri et al., 2013). However, the efficiency of this combined strategy by using MAS within a hexaploid-derived population to rapidly increase resistance allele dosage and achieve effective phenotypic resistance has not been evaluated.

To address these questions, we developed a population of new-type *B. juncea* by employing the hexaploid bridge strategy and advanced it through successive selfing generations. Our study aimed to (i) track fertility restoration over selfing generations, (ii) analyze phenotypic and genomic dynamics, and (iii) demonstrate practical trait improvement by introgressing CR from ECD04 into the *B. juncea* background via MAS. This work not only establishes the hexaploid bridge as an efficient strategy to diversify the *B. juncea* gene pool, but also provides a novel and diverse germplasm resource for *B. juncea* breeding.

## 2 Materials and methods

### 2.1 Plant materials

An effective strategy in developing *B. juncea*-type hybrids, i.e., the hexaploid bridging strategy was developed in our previous study (Mei et al., 2022) and employed in the present study to generate new-type *B. juncea* germplasm. In brief, 32 *B. nigra* accessions were randomly cross-pollinated with three A^j^A^j^A^r^A^r^B^j^B^j^ hexaploid genotypes (designated MS2, MS3 and MS4) to produce A^j^A^r^B^n^B^j^-type F_1_ hybrids, and 20 *B. rapa* accessions were crossed with one A^j^A^j^B^j^B^j^B^n^B^n^ (designated MS6) hexaploid to produce A^j^A^r^B^n^B^j^-type F_1_ hybrids. Resultant F_1_ hybrids were self-pollinated to produce F_2_, F_3_ and other generations. From the above *B. rapa* panel, the European fodder turnip ECD04 (AA) — a known donor harboring multiple resistance genes against *Plasmodiophora brassicae* — was specifically crossed with the hexaploid bridge line MS6 to initiate CR gene transfer. Phenotypic observations (e.g., leaf morphology) and imaging were conducted concurrently at designated growth stages (e.g., seedling stage) to ensure direct comparability across all genotypes. Fertility was assessed by counting the number of seeds per pod from 30 consecutive pods collected from the primary inflorescence of each plant. All materials, including the parental lines (*B. nigra*, *B. rapa*, hexaploids MS2-MS6 and ECD04), the derived new-type lines (F_1_–F_5_), and ten control accessions of current *B. juncea* (vegetable and oil types) from Southwest University (SWU), were sown and transplanted on the same dates in the experimental field at SWU. A uniform planting design was maintained with 30 cm between rows and 25 cm within rows. Detailed information for all accessions and crosses is provided in **Supplementary Table S1**.

### 2.2 Genome resequencing and data analysis

To understand the genetic characteristic of new-type *B. juncea* germplasm, 61 new-type *B. juncea* individuals were randomly selected from the F_1_ and F_2_ generations for whole-genome resequencing, together with 20 *B. nigra* parents, the four hexaploid parents, three *B. rapa* parents, and ten current *B. juncea* accessions. Young leaf samples from these materials were submitted to Wuhan Benagen Biotech Co., Ltd. for whole-genome resequencing using BGISEQ-500. Sequencing yielded an average depth ranging from 9.3× to 16.6× per sample. Following whole-genome resequencing, the genomic sequence data were subjected to quality assessment and filtering using fastp, which included: removal of adapter sequences, trimming of poly G and poly X tails from reads, discarding reads containing more than 5 N bases, removing reads in which the proportion of bases with quality scores below 15 exceeded 40%, eliminating reads with an average base quality score lower than 20, and discarding reads shorter than 50 bp after filtering.

Clean reads were aligned to the reference genome of *B. juncea* (*BjuIR: Brassica juncea* information resource) using BWA. Variants were called using Genome Analysis Toolkit (https://wiki.rc.usf.edu/index.php/Genome_Analysis_ToolKit_GATK), with SNPs and InDels filtered based on specific criteria. Genetic distances among materials were calculated based on the SNPs and InDels using Powermarker V 3.25 (https://brcwebportal.cos.ncsu.edu/powermarker/). Phylogenetic trees were constructed using MEGA software (https://www.megasoftware.net/), and principal component analysis (PCA) was performed using Rstudio.

### 2.3 DNA extraction

Genomic DNA was isolated from fresh young leaf tissues of all plant materials using a modified cetyltrimethylammonium bromide (CTAB) method. Approximately 100 mg of tissue was ground in liquid nitrogen and incubated in pre-warmed CTAB extraction buffer at 65 °C for 45 min. After chloroform: isoamyl alcohol (24:1) extraction, DNA was precipitated with isopropanol, washed with 70% ethanol, and dissolved in nuclease-free water. DNA concentration and quality were assessed using a NanoDrop spectrophotometer and agarose gel electrophoresis.

### 2.4 Marker-assisted selection

To track the introgression of CR genes, genotyping was performed using four PCR-based markers (Powell et al., 1996): CR-m090a (linked to PbBa8.1), CR-BSA3 (linked to PbBa1.1), and CR-S14R14 and CR-S17R17 (both linked to BraA.CR.b) (Chen et al., 2013a; Hirani et al., 2018; primer sequences in **Supplementary Table S2**). All F2 individuals from the cross MS6 × ECD04 were screened using these markers, and those carrying the maximum number of ECD04-specific alleles were self-pollinated Selection was carried out in subsequent generations (F3 to F5) by genotyping ten plants per line with the same markers. PCR was carried out in 10 µL reactions containing approximately 50 ng genomic DNA, 0.5 µL each of forward and reverse primer (10 µM), 5 µL of 2× PCR Master Mix (Vazyme, China), and nuclease-free water to volume. The thermal cycling conditions were as follows: initial denaturation at 94 °C for 5 min; 10 cycles of touchdown PCR (94 °C for 30 s, annealing from 62 °C to 57 °C decreasing by 0.5 °C per cycle for 30 s, 72 °C for 30 s); followed by 25 cycles of standard PCR (94 °C for 30 s, 55 °C for 30 s, 72 °C for 30 s); and a final extension at 72 °C for 5 min. Reactions were held at 16 °C post-amplification. The products were separated by electrophoresis on a 2% agarose gel. Clear bands exceeding the background intensity were scored as 1 (presence of the resistance allele), while faint or absent bands were scored as 0. The ECD04 allele frequency (AF, %) was calculated as.


$$AF=\frac{Np}{Nt}\times 100\%$$

where Np indicates the number of plants identified as positive for the molecular marker, and Nt represents the total number of plants assayed. The line graph depicting ECD04 allele frequencies for each marker was generated using GraphPad Prism.

### 2.5 Clubroot resistance evaluation

To evaluate clubroot resistance, the plants were inoculated with Plasmodiophora brassicae race 4 using the injection method in lab (Jubault et al., 2013) under controlled conditions (22–25 °C, 60% relative humidity). Three replicates were set up for each tested line, with 30 seeds sown per replicate. Disease incidence (DI) and severity were assessed 42 days post-inoculation. Based on root symptoms and gall size, disease severity was classified on a 0–4 scale (Chen et al., 2021), with 0 indicating no symptoms and 4 representing the most severe root deformation. The DI was calculated as follows (Ludwig-Müller et al., 2017):


$$DI=\frac{1\times N1+2\times N2+3\times N3+4\times N4}{4\times Nt}\times 100$$

where N1-N4 indicate the number of plants in each disease severity category, and Nt represents the total number of inoculated plants. The correlation between the DI and the ECD04 AF was calculated and graphically presented using GraphPad Prism.

## 3 Result

### 3.1 Fertility characteristics of new-type *B. juncea*

**Figure 1:**
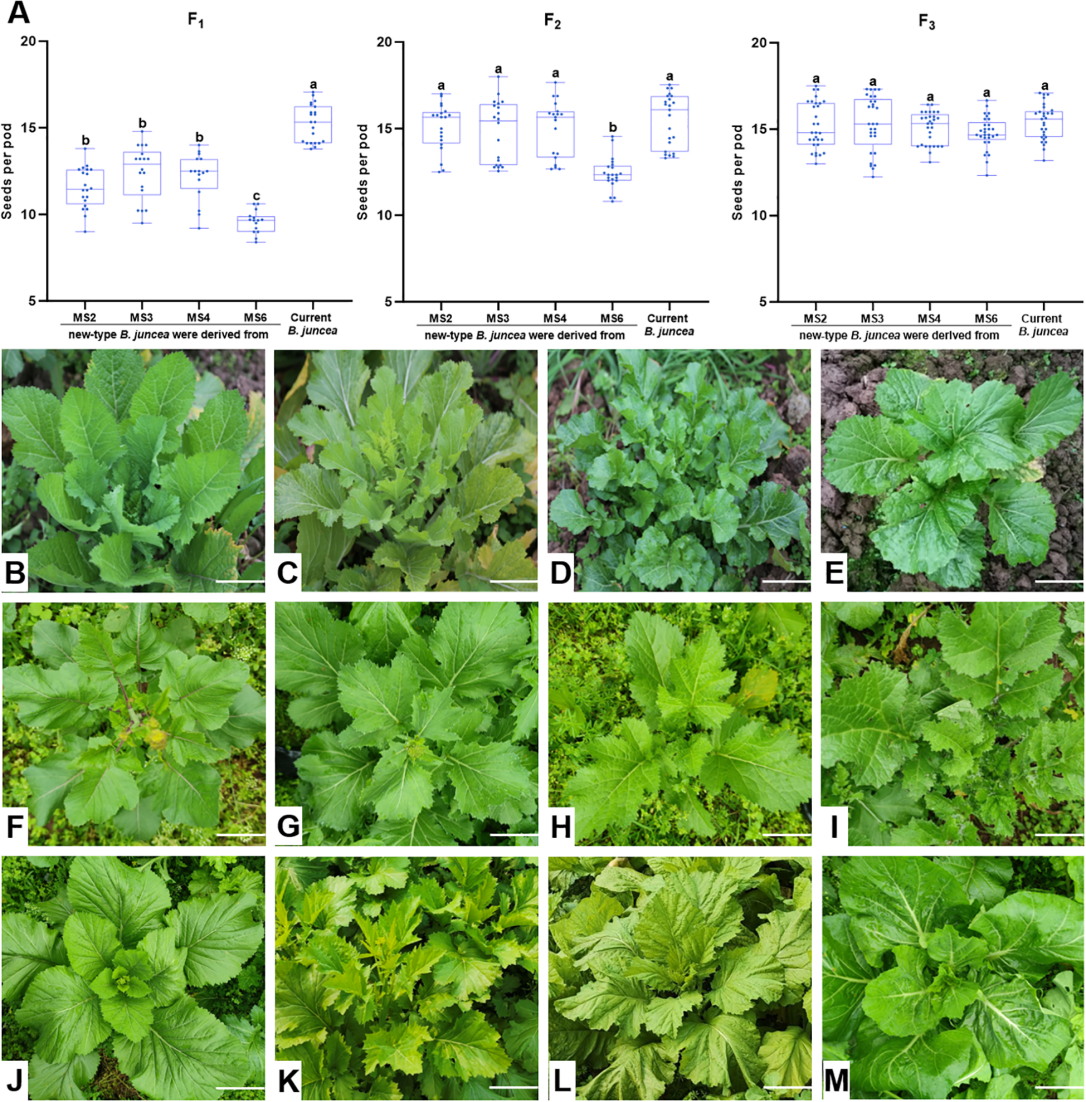
Variation in seeds per pod and phenotypic traits in F_1_, F_2_, and F_3_ new-type *B . juncea*. Different letters above bars indicate significant difference at *p* < 0.05. Images show the variation in leaf shape and color of new-type *B . juncea*; bars =10 cm. **(A)** Seeds per pod in F_1_, F_2_, and F_3_ derived from crosses with different hexaploid parents. MS2 (A^j^A^j^A^r^A^r^B^j^B^j^), MS3 (A^j^A^j^A^r^A^r^B^j^B^j^), MS4 (A^j^A^j^A^r^A^r^B^j^B^j^) and MS6 (A^j^A^j^B^j^B^j^B^n^B^n^) denote the specific hexaploid parent used in each cross combination. **(B-E)** Phenotypic traits in F_1_ plants (images are derived from Mei et al., 2022). **(F, G)** Additional examples of phenotypic traits in F_1_ plants (newly developed in this study). The F_1_ plants of the new-type *B . juncea* exhibited distinct morphological characteristics, including leaf trichomes and purple venation (Figures F) as well as pinnately lobed leaves with prominent venation . **(G-J)** Phenotypic traits in F_2_ plants. In the F_2_ generation, typical F_1_ traits such as trichomes and serrated leaf margins became less pronounced. **(K-M)** Phenotypic traits in F_3_ plants. By the F_3_ generation, a subset of plants converged morphologically toward existing cultivated *B . juncea* types **(K , L)**, while some materials retained the leaf glossiness characteristic of *B . rapa***(M)**.

Through hybridization between hexaploids and diploids, followed by successive selfing, we developed 180 new-type *B. juncea* lines, comprising 76 F_1_ hybrids (from direct crosses), 104 advanced selfing lines (46 F_2_, 26 F_3_, 23 F_4_ and 9 F_5_ generation). To evaluate the fertility of the new-type *B. juncea* lines, we quantified the seed set rate, measured as seeds per pod, for the F_1_, F_2_, and F_3_ generations. Statistical analysis across various generations (**Figure 1A**) revealed that in the F_1_ and F_2_ generations, hybrids derived from MS2, MS3, and MS4 (A^j^A^j^A^r^A^r^B^j^B^j^) exhibited higher seed set rate compared to those derived from MS6 (A^j^A^j^B^j^B^j^B^n^B^n^), suggesting that the different hybridization combinations significantly affect the fertility of hybrids, i.e., the cross of hexaploid A^j^A^j^A^r^A^r^B^j^B^j^ with *B. nigra* is easier to produce hybrids with better fertility in early generations than the cross between hexaploid A^j^A^j^B^j^B^j^B^n^B^n^ and *B. rapa*. More importantly, the data demonstrated that the seed set rate of the hybrids significantly improved with successive generations, particularly noticeable in the hybrids derived from MS6. This indicates that successive selfing can effectively enhance the fertility of all hybrids, regardless of whether they are hybrids of A^j^A^j^A^r^A^r^B^j^B^j^ × A^r^A^r^ or A^j^A^j^B^j^B^j^B^n^B^n^ × B^n^B^n^.

### 3.2 Phenotypic diversity of new-type *B. juncea*

Consistent with fertility restoration, the new-type *B. juncea* lines showed dynamic morphological changes from the F_1_ to F_3_ generations (**Figures 1B-M**). The F_1_ plants exhibited considerable phenotypic variation, including pinnately lobed leaves, leaf trichomes, purple venation and stem, and prominent venation. In the F_2_ generation, while phenotypic variation persisted, a few plants began to converge toward current *B. juncea* traits, with certain F_1_ features such as trichomes and serrated leaf margins becoming less distinct. By the F_3_ generation, some plants clearly converged toward the morphology of current *B. juncea* cultivars. These morphological changes demonstrate that successive selfing generations enabled the selection of plants with a high degree of phenotypic identity to current *B. juncea* from the new-type lines.

### 3.3 Genetic diversity of new-type *B. juncea*

The observed convergence in both fertility and phenotype suggested underlying genetic changes. To elucidate the genetic characteristics of the new-type *B. juncea*, whole-genome resequencing was conducted on the 61 new-type *B. juncea* lines (24 F_1_ and 37 F_2_), alongside 23 parental *B. rapa* and *B. nigra* accessions, 10 current *B. juncea* accessions (including vegetative and oilseed types), and four hexaploid parents. After strict filtering, 24.6 million high-confidence SNPs and small indels were retained, spanning the A and B sub-genomes, with a mean density of 1.36 million variants per chromosome.

**Figure 2:**
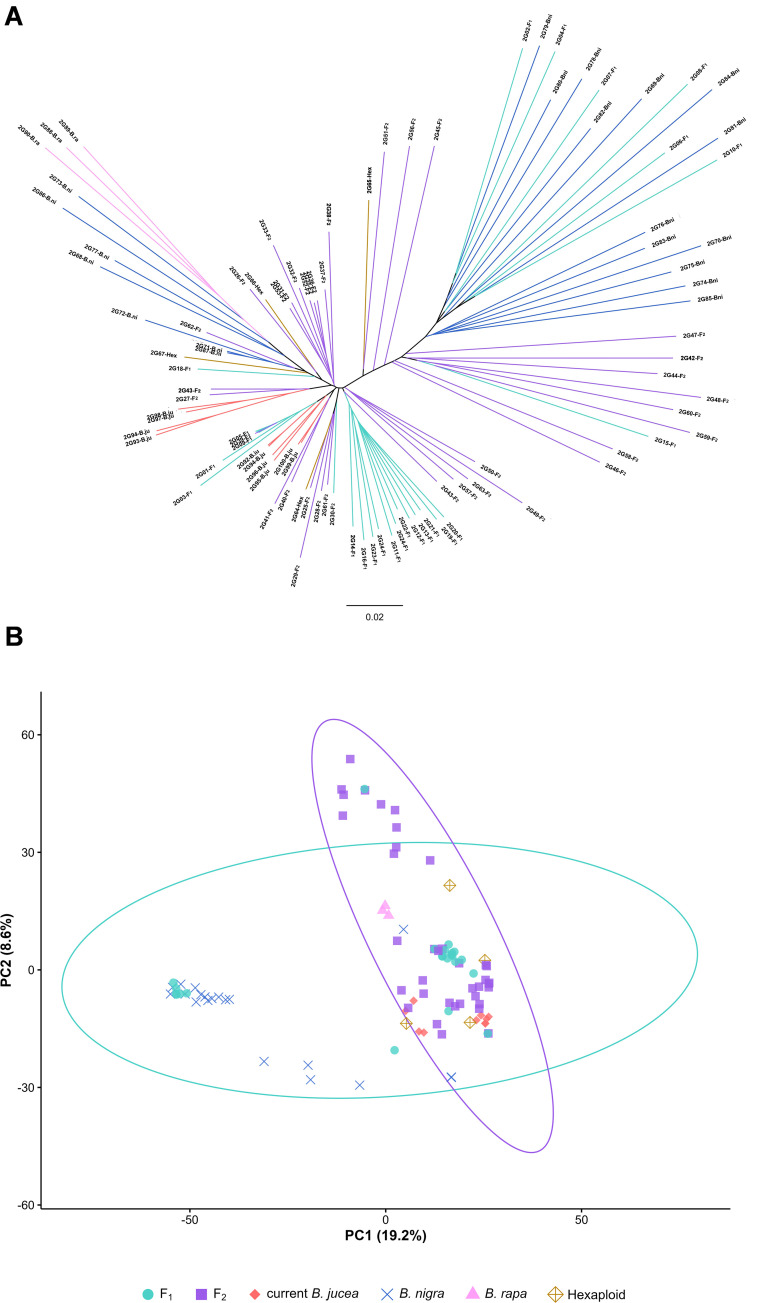
Phylogenomic and principal component analysis results of F_1_and F_2_ new-type *B . juncea*, current *B . juncea*, *B . rapa B . nigra* and hexaploid. The accessions of F_1_, F_2,_ current *B . juncea*, *B . rapa, B . nigra* and hexaploids are denoted by the suffixes F_1_, F_2,_ Bju, Bar, Bni and Hex, respectively. F_1_ represented by teal, F_2_ represented by purple, current *B . jucea* represented by red, *B . nigra* represented by blue, *B . rapa* represented by pink, hexaploid represented by yellow. The F_1_ individuals are genetically distinct and scattered, while the F_2_ individuals show a shift toward the cluster of current *B. juncea*. **(A)** Phylogenomic result; branches in different colors represent different materials. **(B)** Principal component analysis result; different colors and distinct shapes represent different materials. The principal components PC1 and PC2 accounted for 19.2% and 8.6% of the total variance, respectively. The confidence ellipses represent the 95% confidence intervals for the F_1_ and F_2_ populations.

As revealed by phylogenomic analysis (**Figure 2A**), both F_1_ and F_2_ generations exhibited substantial genetic variation as compared to current *B. juncea* accessions. Most F_1_ individuals occupied a long-branch lineage diverging prior to the clade of current *B. juncea*, whereas F_2_ individuals formed a paraphyletic group partially overlapping with current *B. juncea*. Consistent with this phylogenetic pattern, the principal component analysis separated most of the F_1_ progeny from current *B. juncea* along Principal Component 1 (PC1) (19.2% of variance) and Principal Component 1 (PC2) (8.6%), while more F_2_ individuals were notably shifted toward the current *B. juncea* cloud along PC1 (**Figure 2B**).

Genetic distance (GD) analysis further quantified these relationships (**Table 1**). The mean within-group GD was lowest in current *B. juncea* (GD_Cj_ = 0.090), intermediate in the F_2_ (GD_F2_ = 0.152), and highest in the F_1_ (GD_F1_ = 0.168). The between-group GD to current *B. juncea* was slightly lower for the F_2_ (GD_F2-Cj_ = 0.136) than for the F_1_ (GD_F1-Cj_ = 0.146), suggesting that the F_2_ lines are still markedly different from current *B. juncea.* However, it also suggests that genetic recombination had already produced some individuals with genomic compositions more closely aligned with current *B. juncea*. Collectively, these results confirm that the new-type lines constitute a unique genetic group, harboring a spectrum of diversity that includes both distinct lineages and F_2_ individuals with genomic compositions closer to current *B. juncea*.

**Table 1:** CD within and among F_1_and F_2_ new-type *B. juncea*, current *B. juncea*, *B. rapa B. nigra* and hexaploid.

Material	*B. juncea*	*B. nigra*	*B. rapa*	Hexaploid	F_1_	F_2_
*B. juncea*	0.090 ± 0.024 h^#^					
*B. nigra*	0.194 ± 0.047 d	0.238 ± 0.054 b				
*B. rapa*	0.199 ± 0.006 cd	0.327 ± 0.009 a	0.172 ± 0.006 e			
Hexaploid	0.153 ± 0.055 f	0.201 ± 0.047 cd	0.209 ± 0.027 c	0.125 ± 0.022 g		
F_1_	0.146 ± 0.055 f	0.217 ± 0.046 c	0.235 ± 0.073 b	0.144 ± 0.052 fg	0.168 ± 0.063 e	
F_2_	0.136 ± 0.052 g	0.212 ± 0.046 c	0.236 ± 0.082 b	0.134 ± 0.048 g	0.169 ± 0.065 e	0.152 ± 0.049 f

^#^ Different letters following values in each column indicate significant difference at *p<* 0.05

### 3.4 Development of clubroot resistant *B. juncea*

To validate the practical application value of the hexaploid bridge hybridization strategy for trait improvement, we adopted a MAS in the hybrids between 6MS6 (A^j^A^j^B^j^B^j^B^n^B^n^) with the European fodder turnip ECD04 which is well known *B. rapa* accession harboring multiple CR loci. Four PCR-based markers tightly linked to these CR loci were assayed in F_2_-F_5_ progeny (**Supplementary Table S3**), and plants carrying the highest number of ECD04-alleles were advanced to the next generation.

**Figure 3:**
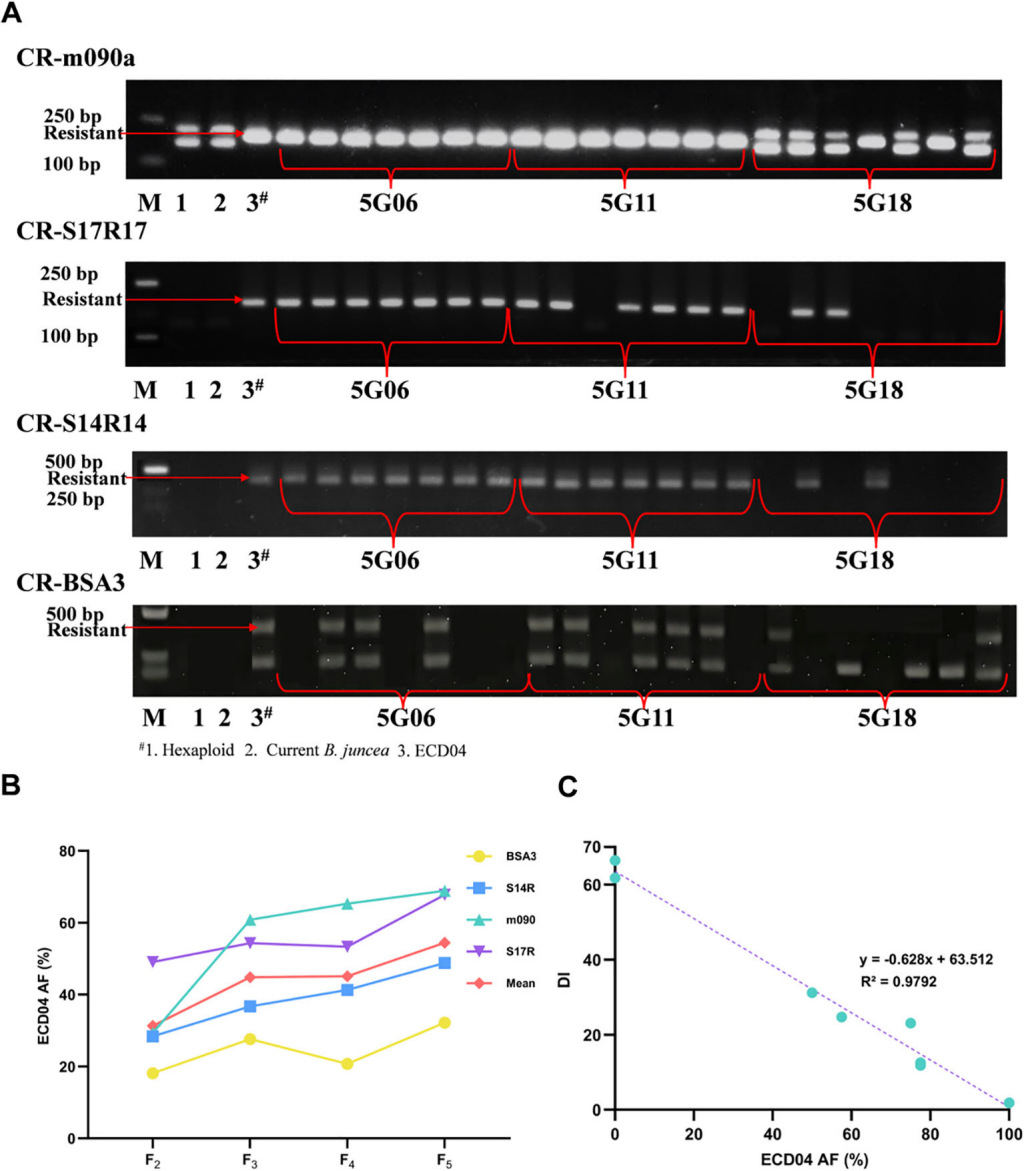
Changes in ECD04 allele frequency (AF, %) across generations, its negative correlation with disease resistance and genotyping of the four CR-linked markers. **(A)** Agarose gel electrophoresis images of PCR amplification using four CR-linked markers for parental and F_5_ individuals. M: DNA molecular weight standard. Arrows indicate the specific bands corresponding to the resistance alleles. The resistance alleles were assigned based on previously reported linkage: markers CR−m090a and CR−BSA3 are linked to the resistance genes *PbBa8.1* and *PbBa1.1*, respectively (Chen et al., 2013a); markers CR−S14R14 and CR−S17R17 are linked to the resistance locus *BraA.CR.b* (Hirani et al., 2018). The resistance−donor parent ECD04 is a homozygous resistant accession: for markers CR−m090a, CR−S14R14 and CR−S17R17 it shows only a single band (the resistance allele); for marker CR−BSA3, although multiple bands were amplified, the arrow points to the target resistance band. Lane labels 5G06, 5G11 and 5G18 denote F_5_ families. Each lane contains DNA from a single plant that was randomly selected from the corresponding family. **(B)** Changes in ECD04 AF of different markers across generations. **(C)** The negative linear correlation between ECD04 AF and DI; *r* = -0.98, *p* < 0.0001.

According to the four CR-linked markers (**Figure 3A**), a progressive enrichment was presented since the mean frequency of ECD04-alleles rose from 31.3% in the F_2_ to 54.4% in the F_5_, representing a 73.8% rise over the selection period (**Figure 3B**). More than half of the F_5_ families (e.g., 5G06, 5G09, 5G10, 5G11 and 5G13) retained ≥ 50% donor alleles, while a subset (5G16-5G19) still carried< 50% ECD04 alleles (**Table 2**). Laboratory phenotyping for CR was carried out on F_5_ lines with AF ≥ 50%, alongside parental controls. A strong negative linear correlation was observed between ECD04 AF and disease index (*r* = -0.98, *p* < 0.0001; **Figure 3C**), indicating that the allele dosage is a reliable predictor of resistance levels.

**Table 2:** Molecular markers and disease incidence statistics of clubroot in F_5_ new-type *B. juncea*.

Marker material	BSA3	S14R14	m090a	S17R17	CR allele frequency (%)	Incidence rate (%)	Disease index
Current *B. juncea*	0/10^*^	0/10	0/10	0/10	0	100 a	66.4 a^#^
Hexaploid MS6	0/10	0/10	0/10	0/10	0	98 a	61.8 a
ECD04	10/10	10/10	10/10	10/10	100	4.30 e	1.90 e
5G06	3/10	10/10	10/10	8/10	77.5	34.4 d	11.8 d
5G09	0/10	6/10	7/10	7/10	50	75.3 b	31.2 b
5G10	0/10	6/10	8/10	9/10	57.5	66.3 c	24.7 c
5G11	5/10	9/10	10/10	7/10	77.5	32 d	12.5 d
5G13	6/10	6/10	8/10	10/10	75	63.3 c	23.1 c
5G16	2/10	3/10	6/10	5/10	40	**-**	**-**
5G17	0/10	3/10	3/10	4/10	25	**-**	**-**
5G18	6/10	0/10	4/10	4/10	35	**-**	**-**
5G19	5/10	1/10	6/10	7/10	47.5	**-**	**-**

^*^ Number of individual plants carrying ECD04-allele/the total plants tested

^#^ Different letters in each column indicate significant difference at *p<* 0.05; ‘**-**’ represents not tested

**Figure 4:**
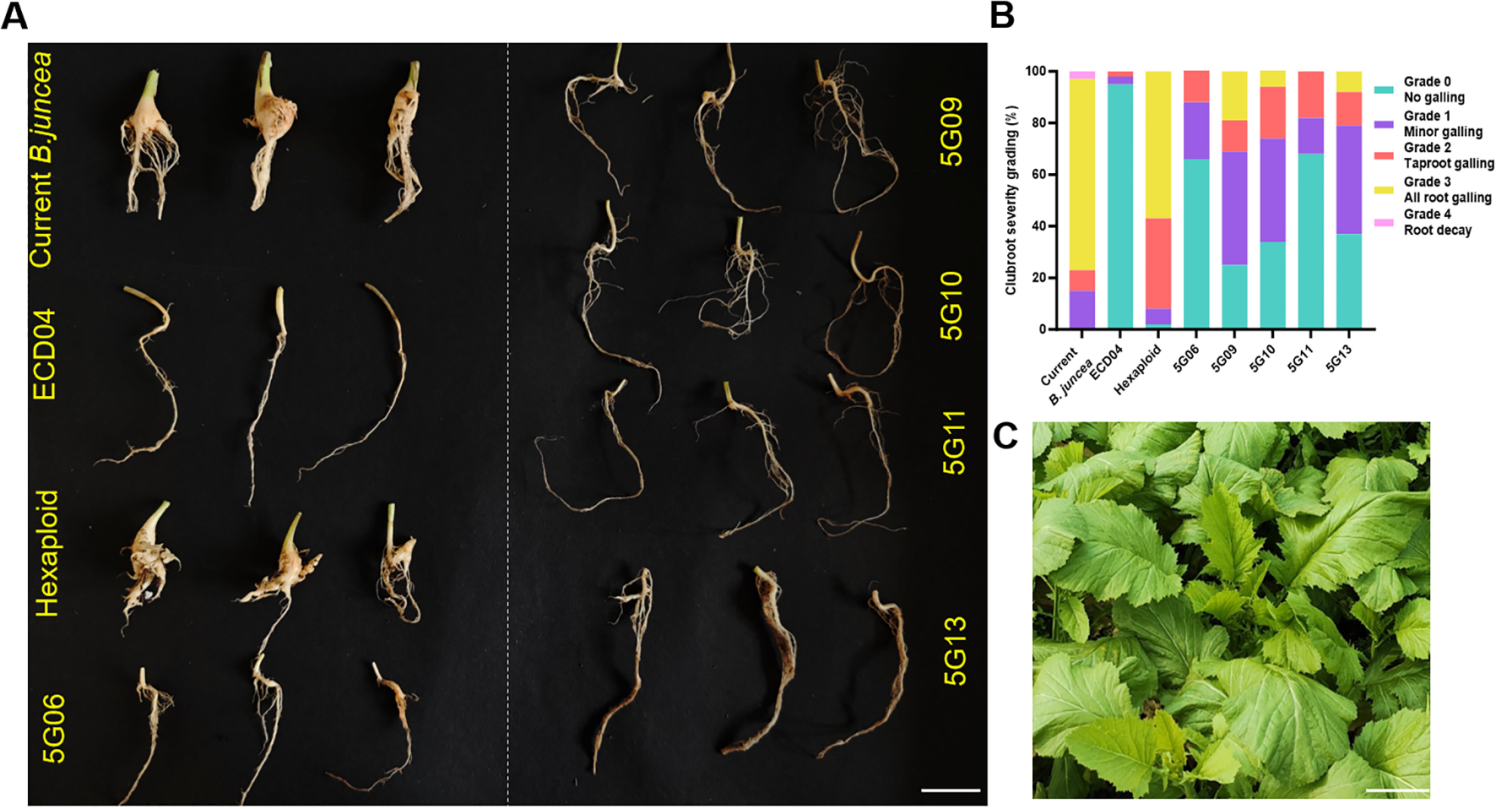
Lab-inoculation results and field phenotype of F_5_ new-type *B . juncea*. **(A)** Phenotyping of clubroot inoculation. Current *B . juncea* and hexaploid were susceptible controls and ECD04 served as the resistant control; inoculated with *Plasmodiophora brassicae* race 4 for 42 d, bar = 10 cm. **(B)** Disease severity grading distribution. Based on the severity of root symptoms, the disease rating scale is divided into 5 grades: grade 0 indicates no symptoms, grade 4 represents the most severe infection and different colors represent different disease grades. **(C)** Field phenotype of F_5_ family 5G06 at seedling stage, bar = 10 cm.

Concerning the detailed resistance (**Figure 4**, **Table 2**), the hexaploid parent and current *B. juncea* exhibited nearly 100% incidence and DI > 60, whereas the resistant parent ECD04 showed near immunity (disease incidence = 4.3%, DI = 1.7). Two F_5_ families with AF = 77.5% (5G06 and 5G11) were virtually homozygous for the target haplotype (especially markers S14R14 and m090a) and recorded the lowest DI values (11.5 and 12.5, respectively), indicating the successful introgression and concentration of ECD04-derived CR loci into the *B. juncea* background. Three other F_5_ families with AF between 50% and 75% displayed moderate resistance, with DI of 23.1-31.2 and disease incidence of 63%-75%, indicating the need for further selfing to achieve full homozygosity.

## 4 Discussion

### 4.1 New-type *B. juncea*, a novel genetic resource for *B. juncea* breeding

The strong reproductive barriers that impede traditional breeding, such as hybrid sterility and embryo abortion, have severely restricted the introgression of novel alleles from diploid progenitors into the cultivated *B. juncea* gene pool (Liu et al., 2018; Zhu et al., 2022). In this study, synthetic hexaploids (AABBBB/AAAABB), proposed as a bridge for gene transfer (Mei et al., 2022), were employed successfully to achieve efficient introgression of genomic components from both progenitor species into *B. juncea*. The newly developed materials represent a distinct and unique gene pool, not merely a random genetic mixture.

Phylogenetic, principal component, and genetic distance analyses consistently demonstrated that the new-type lines, particularly the F_1_ generation, formed a cluster separate from natural *B. juncea*. Although a genetic shift toward natural *B. juncea* was observed in the F_2_ generation, the genetic distance between the new-type lines and natural *B. juncea* remained substantially larger than that within natural *B. juncea* itself. These lines therefore retain a unique set of alleles from the progenitor species, collectively establishing a valuable and novel genetic reservoir. This population can serve as a diverse foundation set for future gene discovery and breeding, directly addressing the issue of genetic constriction in current cultivars.

### 4.2 Rapid fertility restoration and genomic stabilization through selfing

The practical utilization of this novel gene pool first requires overcoming a common challenge in newly synthesized polyploids, i.e., meiotic instability, which often leads to reduced fertility (Tian et al., 2023). The significantly lower seeds per pod in the F_1_ generation in our study confirmed such instability, reflecting a form of genomic shock characterized by rapid genomic reorganization in response to aberrant homologous chromosome pairing (McClintock, 1984; Xiong et al., 2011; Martin and Jouve, 1992). Nevertheless, the fertility was rapidly restored in subsequent generations. This recovery is consistent with a similar scenario (Xiong et al., 2011), wherein meiotic stability is gradually established through restricted homologous pairing, epigenetic silencing of conflict regions, and selective elimination of cytologically unstable lineages. Concurrently, phenotypic convergence from diploid-parent-like morphological variation in the F_1_ to current *B. juncea* type traits in the F_3_ was possibly also driven by recurrent selfing. The increase in homozygosity through selfing not only facilitated stable chromosome pairing but also purged deleterious genomic interactions, thereby accelerating the emergence of a cytologically and phenotypically stable allopolyploid.

### 4.3 Efficient trait introgression and a clear dosage effect of CR loci

Building upon this rapid stabilization, MAS was effectively applied within the complex polyploid background. Four cycles of targeted selection substantially enriched CR alleles from ECD04, demonstrating the high efficiency and precision of the hexaploid bridge strategy for trait improvement. Notably, a strong linear relationship was observed between allelic dosage of the resistance loci and disease severity, indicating that the introgressed CR genes likely act in a dominant or additive manner (Li et al., 2016). This relationship enabled reliable phenotypic prediction in early generations based on genotypic data, significantly improving selection efficiency.

However, our findings also reveal a limitation of the dosage model. Family 5G13, which was fixed for the major locus S17 and carried 75% donor alleles across the four target loci, still exhibited 63% disease incidence. We hypothesize that the following factors may be responsible: (i) linkage drag, in which undesirable genomic segments tightly linked to the target CR loci were co-introgressed, potentially carrying alleles that compromise resistance or plant fitness (Jabran et al., 2023); (ii) residual heterozygosity at loci BSA3, S14, and m090a (87.5% donor alleles, not yet fixed), or the presence of tightly linked deleterious segments near S17, may have compromised the expected resistance; (iii) background effects from the recipient *B. juncea* genome, where interactions between the introgressed alleles and the genetic background may modulate the final resistance level. This case illustrates a general limitation of relying solely on foreground selection in marker-assisted breeding (Dakouri et al., 2013). Therefore, incorporating routine background selection is recommended to break such unfavorable linkages and accelerate recovery of the recurrent parent genome (Nannuru et al., 2025), ultimately facilitating the development of agronomically superior lines with stable and complete clubroot resistance.

## 5 Conclusion and perspectives

In summary, this study establishes an efficient framework for polyploid crop improvement via synthetic hexaploid bridges. The substantial genetic diversity within this novel germplasm pool created in this study offers a promising resource for discovering alleles underlying valuable agronomic traits from *B. rapa* and *B. nigra*. This strategy not only broadens the genetic base of *B. juncea* but also enables precision breeding for clubroot resistance. Future efforts will focus on multi-environment field evaluations of advanced lines to assess their yield potential and adaptability, paving the way for commercial deployment.

## Data Availability

The raw whole-genome sequencing data generated in this study have been deposited, in accordance with the journal’s requirements, in the BIG Submission database of the National Genomics Data Center (NGDC) in China. The data can be accessed via the BioProject accession number PRJCA049343 at the following link: https://ngdc.cncb.ac.cn/bioproject/browse/PRJCA049343.
