# Supplementary material for: Hexaploid-bridged introgression broadens genetic diversity and enriches clubroot-resistance alleles in Brassica juncea
Source: Front Plant Sci. 2026 Jan 22;16:1729594. doi: 10.3389/fpls.2025.1729594 (PMC12872742; doi:10.3389/fpls.2025.1729594)
Supplement: Supplementary file 1 [file DataSheet1.pdf]

Table S1 All plant materials used in this study were assigned the most up-to-date numbering system currently in use

Code	Species	Accession	Origin	Generation
2G64	Hexaploid	6MS2-2	SWU	-
2G65	Hexaploid	6MS3-2	SWU	-
2G66	Hexaploid	6MS4-2	SWU	-
2G67	Hexaploid	6MS6-2	SWU	-
2G68	*B. nigra*	PI131512	India, Uttar Pradesh	-
2G69	*B. nigra*	PI179846	Turkey	-
2G70	*B. nigra*	PI179851	-	-
2G71	*B. nigra*	PI209782	India	-
2G72	*B. nigra*	PI220282	Netherlands	-
2G73	*B. nigra*	PI273639	Idia,Punjab	-
2G74	*B. nigra*	PI357368	Idia,Punjab	-
2G75	*B. nigra*	PI459012	Germany	-
2G76	*B. nigra*	PI459013	Afghanistan,Kabul	-
2G77	*B. nigra*	Ames15655	United States	-
2G78	*B. nigra*	PI174795	Monteneg	-
2G79	*B. nigra*	PI347620	India	-
2G80	*B. nigra*	PI347621	California	-
2G81	*B. nigra*	PI357370	Idia	-
2G82	*B. nigra*	PI426409	India	-
2G83	*B. nigra*	PI458981	Pakistan	-
2G84	*B. nigra*	PI459011	United States	-
2G85	*B. nigra*	PI603019	India	-
2G86	*B. nigra*	PI633148	Italy	-
2G87	*B. nigra*	PI649156	NSSL280414 51 SD	-
1G01	*B. nigra*	PI195554	Ethiopia	-
1G02	*B. nigra*	PI273639	Germany	-
1G03	*B. nigra*	PI194902	Idia	-
1G04	*B. nigra*	PI197401	India, Uttar Pradesh	-
1G05	*B. nigra*	PI248770	Germany	-
1G06	*B. nigra*	PI273642	India	-
1G07	*B. nigra*	PI280638	Turkey	-
1G08	*B. nigra*	PI173860	Pakistan	-
1G09	*B. nigra*	PI633143	Iraq	-
1G10	*B. nigra*	PI633146	Afghanistan,Kabul	-
1G11	*B. nigra*	K003	-	-
1G12	*B. nigra*	K006	-	-
2G88	*B. rapa*	PI 458931	Sweden	-
2G89	*B. rapa*	PI250004	Egypt	-
2G90	*B. rapa*	PI254540	Iraq	-

Code	Species	Accession	Origin	Generation
1G13	*B. rapa*	PI256054	Afghanistan	-
1G14	*B. rapa*	PI267726	Japan, Kyoto	-
1G15	*B. rapa*	PI458614	New Zealand	-
1G16	*B. rapa*	PI 458977	United States	-
1G17	*B. rapa*	PI 458932	Sweden	-
1G18	*B. rapa*	PI 458933	Sweden	-
1G19	*B. rapa*	PI 603026	Pakistan	-
1G20	*B. rapa*	PI 458982	India	-
1G21	*B. rapa*	ECD4	Europe	-
1G22	*B. rapa*	PI 633153	France	-
1G23	*B. rapa*	PI 633159	Korea, South	-
1G24	*B. rapa*	8003	China	-
1G25	*B. rapa*	8004	China	-
1G26	*B. rapa*	8007	China	-
1G27	*B. rapa*	8008	China	-
1G28	*B. rapa*	8010	China	-
1G29	*B. rapa*	8011	China	-
2G91	current *B. juncea*	-	SWU	-
2G92	current *B. juncea*	-	SWU	-
2G93	current *B. juncea*	-	SWU	-
2G94	current *B. juncea*	-	SWU	-
2G95	current *B. juncea*	-	SWU	-
2G96	current *B. juncea*	-	SWU	-
2G97	current *B. juncea*	-	SWU	-
2G98	current *B. juncea*	-	SWU	-
2G99	current *B. juncea*	-	SWU	-
2G100	current *B. juncea*	-	SWU	-
1G30	new-type *B. juncea*	2G64×1G01	Hybrid Synthesis	F_1
1G31	new-type *B. juncea*	2G64×1G02	Hybrid Synthesis	F_1
1G32	new-type *B. juncea*	2G64×1G03	Hybrid Synthesis	F_1
1G33	new-type *B. juncea*	2G64×1G04	Hybrid Synthesis	F_1
1G34	new-type *B. juncea*	2G64×1G05	Hybrid Synthesis	F_1
1G35	new-type *B. juncea*	2G64×1G06	Hybrid Synthesis	F_1
1G36	new-type *B. juncea*	2G64×1G07	Hybrid Synthesis	F_1
1G37	new-type *B. juncea*	2G64×1G08	Hybrid Synthesis	F_1
1G38	new-type *B. juncea*	2G64×1G09	Hybrid Synthesis	F_1
1G39	new-type *B. juncea*	2G64×1G10	Hybrid Synthesis	F_1
1G40	new-type *B. juncea*	2G64×1G11	Hybrid Synthesis	F_1
1G41	new-type *B. juncea*	2G64×1G12	Hybrid Synthesis	F_1
1G42	new-type *B. juncea*	2G64×1G13	Hybrid Synthesis	F_1
1G43	new-type *B. juncea*	2G64×1G14	Hybrid Synthesis	F_1
1G44	new-type *B. juncea*	2G64×1G15	Hybrid Synthesis	F_1
1G45	new-type *B. juncea*	2G64×1G16	Hybrid Synthesis	F_1

Code	Species	Accession	Origin	Generation
1G46	new-type *B. juncea*	2G64×1G17	Hybrid Synthesis	F_1
1G47	new-type *B. juncea*	2G67×1G13	Hybrid Synthesis	F_1
1G48	new-type *B. juncea*	2G67×1G14	Hybrid Synthesis	F_1
1G49	new-type *B. juncea*	2G67×1G15	Hybrid Synthesis	F_1
1G50	new-type *B. juncea*	2G67×1G16	Hybrid Synthesis	F_1
1G51	new-type *B. juncea*	2G67×1G17	Hybrid Synthesis	F_1
1G52	new-type *B. juncea*	2G67×1G18	Hybrid Synthesis	F_1
1G53	new-type *B. juncea*	2G67×1G19	Hybrid Synthesis	F_1
1G54	new-type *B. juncea*	2G67×1G20	Hybrid Synthesis	F_1
1G55	new-type *B. juncea*	2G67×1G21	Hybrid Synthesis	F_1
1G56	new-type *B. juncea*	2G67×1G22	Hybrid Synthesis	F_1
1G57	new-type *B. juncea*	2G67×1G23	Hybrid Synthesis	F_1
1G58	new-type *B. juncea*	2G67×1G24	Hybrid Synthesis	F_1
1G59	new-type *B. juncea*	2G67×1G25	Hybrid Synthesis	F_1
1G60	new-type *B. juncea*	2G67×1G26	Hybrid Synthesis	F_1
1G61	new-type *B. juncea*	2G67×1G27	Hybrid Synthesis	F_1
1G62	new-type *B. juncea*	2G67×1G28	Hybrid Synthesis	F_1
1G63	new-type *B. juncea*	2G67×1G29	Hybrid Synthesis	F_1
1G64	new-type *B. juncea*	2G65×1G01	Hybrid Synthesis	F_1
1G65	new-type *B. juncea*	2G65×1G02	Hybrid Synthesis	F_1
1G66	new-type *B. juncea*	2G65×1G03	Hybrid Synthesis	F_1
1G67	new-type *B. juncea*	2G65×1G04	Hybrid Synthesis	F_1
1G68	new-type *B. juncea*	2G65×1G05	Hybrid Synthesis	F_1
1G69	new-type *B. juncea*	2G65×1G06	Hybrid Synthesis	F_1
1G70	new-type *B. juncea*	2G65×1G07	Hybrid Synthesis	F_1
1G71	new-type *B. juncea*	2G65×1G08	Hybrid Synthesis	F_1
1G72	new-type *B. juncea*	2G65×1G09	Hybrid Synthesis	F_1
1G73	new-type *B. juncea*	2G66×1G01	Hybrid Synthesis	F_1
1G74	new-type *B. juncea*	2G66×1G02	Hybrid Synthesis	F_1
1G75	new-type *B. juncea*	2G66×1G03	Hybrid Synthesis	F_1
2G01	new-type *B. juncea*	2G64×1G01	Hybrid Synthesis	F_1
2G02	new-type *B. juncea*	2G64×1G02	Hybrid Synthesis	F_1
2G03	new-type *B. juncea*	2G64×1G03	Hybrid Synthesis	F_1
2G04	new-type *B. juncea*	2G64×1G04	Hybrid Synthesis	F_1
2G05	new-type *B. juncea*	2G65×1G05	Hybrid Synthesis	F_1
2G06	new-type *B. juncea*	2G65×1G06	Hybrid Synthesis	F_1
2G07	new-type *B. juncea*	2G65×1G07	Hybrid Synthesis	F_1
2G08	new-type *B. juncea*	2G65×1G08	Hybrid Synthesis	F_1
2G09	new-type *B. juncea*	2G66×1G09	Hybrid Synthesis	F_1
2G10	new-type *B. juncea*	2G66×1G10	Hybrid Synthesis	F_1
2G11	new-type *B. juncea*	2G66×1G11	Hybrid Synthesis	F_1
2G12	new-type *B. juncea*	2G66×1G12	Hybrid Synthesis	F_1
2G13	new-type *B. juncea*	2G66×1G13	Hybrid Synthesis	F_1

Code	Species	Accession	Origin	Generation
2G14	new-type *B. juncea*	2G67×2G87	Hybrid Synthesis	F_1
2G15	new-type *B. juncea*	2G67×2G88	Hybrid Synthesis	F_1
2G16	new-type *B. juncea*	2G67×2G89	Hybrid Synthesis	F_1
2G17	new-type *B. juncea*	2G67×2G90	Hybrid Synthesis	F_1
2G18	new-type *B. juncea*	2G67×2G91	Hybrid Synthesis	F_1
2G19	new-type *B. juncea*	2G67×2G92	Hybrid Synthesis	F_1
2G20	new-type *B. juncea*	2G67×2G93	Hybrid Synthesis	F_1
2G21	new-type *B. juncea*	2G67×2G94	Hybrid Synthesis	F_1
2G22	new-type *B. juncea*	2G67×2G95	Hybrid Synthesis	F_1
2G23	new-type *B. juncea*	2G67×2G96	Hybrid Synthesis	F_1
2G24	new-type *B. juncea*	2G67×2G97	Hybrid Synthesis	F_1
3G19	new-type *B. juncea*	2G64×2G67	Hybrid Synthesis	F_1
3G20	new-type *B. juncea*	2G64×2G68	Hybrid Synthesis	F_1
3G21	new-type *B. juncea*	2G65×2G68	Hybrid Synthesis	F_1
3G22	new-type *B. juncea*	2G67×2G87	Hybrid Synthesis	F_1
3G23	new-type *B. juncea*	2G67×2G88	Hybrid Synthesis	F_1
3G24	new-type *B. juncea*	2G65×2G68	Hybrid Synthesis	F_1
2G25	new-type *B. juncea*	1G47	Hybrid Synthesis	F_2
2G26	new-type *B. juncea*	1G47	Hybrid Synthesis	F_2
2G27	new-type *B. juncea*	1G48	Hybrid Synthesis	F_2
2G28	new-type *B. juncea*	1G49	Hybrid Synthesis	F_2
2G29	new-type *B. juncea*	1G50	Hybrid Synthesis	F_2
2G30	new-type *B. juncea*	1G51	Hybrid Synthesis	F_2
2G31	new-type *B. juncea*	1G52	Hybrid Synthesis	F_2
2G32	new-type *B. juncea*	1G53	Hybrid Synthesis	F_2
2G33	new-type *B. juncea*	1G54	Hybrid Synthesis	F_2
2G34	new-type *B. juncea*	1G56	Hybrid Synthesis	F_2
2G35	new-type *B. juncea*	1G57	Hybrid Synthesis	F_2
2G36	new-type *B. juncea*	1G58	Hybrid Synthesis	F_2
2G37	new-type *B. juncea*	1G59	Hybrid Synthesis	F_2
2G38	new-type *B. juncea*	1G60	Hybrid Synthesis	F_2
2G39	new-type *B. juncea*	1G61	Hybrid Synthesis	F_2
2G40	new-type *B. juncea*	1G62	Hybrid Synthesis	F_2
2G41	new-type *B. juncea*	1G63	Hybrid Synthesis	F_2
2G42	new-type *B. juncea*	1G64	Hybrid Synthesis	F_2
2G43	new-type *B. juncea*	1G65	Hybrid Synthesis	F_2
2G44	new-type *B. juncea*	1G66	Hybrid Synthesis	F_2
2G45	new-type *B. juncea*	1G67	Hybrid Synthesis	F_2
2G46	new-type *B. juncea*	1G30	Hybrid Synthesis	F_2
2G47	new-type *B. juncea*	1G31	Hybrid Synthesis	F_2
2G48	new-type *B. juncea*	1G32	Hybrid Synthesis	F_2
2G49	new-type *B. juncea*	1G33	Hybrid Synthesis	F_2
2G50	new-type *B. juncea*	1G34	Hybrid Synthesis	F_2

Code	Species	Accession	Origin	Generation
2G51	new-type *B. juncea*	1G35	Hybrid Synthesis	F_2
2G52	new-type *B. juncea*	1G36	Hybrid Synthesis	F_2
2G53	new-type *B. juncea*	1G55	Hybrid Synthesis	F_2
2G54	new-type *B. juncea*	1G55	Hybrid Synthesis	F_2
2G55	new-type *B. juncea*	1G55	Hybrid Synthesis	F_2
2G56	new-type *B. juncea*	1G40	Hybrid Synthesis	F_2
2G57	new-type *B. juncea*	1G41	Hybrid Synthesis	F_2
2G58	new-type *B. juncea*	1G42	Hybrid Synthesis	F_2
2G59	new-type *B. juncea*	1G43	Hybrid Synthesis	F_2
2G60	new-type *B. juncea*	1G44	Hybrid Synthesis	F_2
2G61	new-type *B. juncea*	1G45	Hybrid Synthesis	F_2
2G62	new-type *B. juncea*	1G46	Hybrid Synthesis	F_2
2G63	new-type *B. juncea*	1G47	Hybrid Synthesis	F_2
3G25	new-type *B. juncea*	2G16	Hybrid Synthesis	F_2
3G26	new-type *B. juncea*	2G17	Hybrid Synthesis	F_2
3G27	new-type *B. juncea*	2G18	Hybrid Synthesis	F_2
3G28	new-type *B. juncea*	2G19	Hybrid Synthesis	F_2
3G29	new-type *B. juncea*	2G20	Hybrid Synthesis	F_2
3G30	new-type *B. juncea*	2G21	Hybrid Synthesis	F_2
3G31	new-type *B. juncea*	2G22	Hybrid Synthesis	F_2
3G32	new-type *B. juncea*	2G33	Hybrid Synthesis	F_3
3G33	new-type *B. juncea*	2G34	Hybrid Synthesis	F_3
3G34	new-type *B. juncea*	2G35	Hybrid Synthesis	F_3
3G35	new-type *B. juncea*	2G36	Hybrid Synthesis	F_3
3G36	new-type *B. juncea*	2G37	Hybrid Synthesis	F_3
3G37	new-type *B. juncea*	2G38	Hybrid Synthesis	F_3
3G38	new-type *B. juncea*	2G39	Hybrid Synthesis	F_3
3G39	new-type *B. juncea*	2G40	Hybrid Synthesis	F_3
3G40	new-type *B. juncea*	2G41	Hybrid Synthesis	F_3
3G42-1	new-type *B. juncea*	2G54	Hybrid Synthesis	F_3
3G42-2	new-type *B. juncea*	2G54	Hybrid Synthesis	F_3
3G42-3	new-type *B. juncea*	2G54	Hybrid Synthesis	F_3
3G42-4	new-type *B. juncea*	2G54	Hybrid Synthesis	F_3
3G42-5	new-type *B. juncea*	2G54	Hybrid Synthesis	F_3
3G42-6	new-type *B. juncea*	2G54	Hybrid Synthesis	F_3
3G42-7	new-type *B. juncea*	2G55	Hybrid Synthesis	F_3
3G47-2	new-type *B. juncea*	2G55	Hybrid Synthesis	F_3
3G47-3	new-type *B. juncea*	2G55	Hybrid Synthesis	F_3
3G47-4	new-type *B. juncea*	2G55	Hybrid Synthesis	F_3
3G47-5	new-type *B. juncea*	2G55	Hybrid Synthesis	F_3
3G47-6	new-type *B. juncea*	2G55	Hybrid Synthesis	F_3
3G47-7	new-type *B. juncea*	2G55	Hybrid Synthesis	F_3
3G49-1	new-type *B. juncea*	2G53	Hybrid Synthesis	F_3

Code	Species	Accession	Origin	Generation
3G49-2	new-type *B. juncea*	2G53	Hybrid Synthesis	F_3
3G49-3	new-type *B. juncea*	2G53	Hybrid Synthesis	F_3
3G49-4	new-type *B. juncea*	2G53	Hybrid Synthesis	F_3
4G01-1	new-type *B. juncea*	3G42-3	Hybrid Synthesis	F_4
4G02-1	new-type *B. juncea*	3G42-4	Hybrid Synthesis	F_4
4G03-1	new-type *B. juncea*	3G47-4	Hybrid Synthesis	F_4
4G04-1	new-type *B. juncea*	3G49-4	Hybrid Synthesis	F_4
4G05-1	new-type *B. juncea*	3G47-3	Hybrid Synthesis	F_4
4G07-1	new-type *B. juncea*	3G47-2	Hybrid Synthesis	F_4
4G12-1	new-type *B. juncea*	3G42-6	Hybrid Synthesis	F_4
4G14-1	new-type *B. juncea*	3G47-5	Hybrid Synthesis	F_4
4G15-1	new-type *B. juncea*	3G47-7	Hybrid Synthesis	F_4
4G158-1	new-type *B. juncea*	3G49-1	Hybrid Synthesis	F_4
4G158-2	new-type *B. juncea*	3G49-1	Hybrid Synthesis	F_4
4G158-3	new-type *B. juncea*	3G49-1	Hybrid Synthesis	F_4
4G158-4	new-type *B. juncea*	3G49-1	Hybrid Synthesis	F_4
4G158-5	new-type *B. juncea*	3G49-1	Hybrid Synthesis	F_4
4G159-1	new-type *B. juncea*	3G49-1	Hybrid Synthesis	F_4
4G159-2	new-type *B. juncea*	3G49-1	Hybrid Synthesis	F_4
4G159-3	new-type *B. juncea*	3G49-1	Hybrid Synthesis	F_4
4G160-1	new-type *B. juncea*	3G49-3	Hybrid Synthesis	F_4
4G16-6	new-type *B. juncea*	3G47-6	Hybrid Synthesis	F_4
4G161-5	new-type *B. juncea*	3G42-1	Hybrid Synthesis	F_4
4G162-1	new-type *B. juncea*	3G42-2	Hybrid Synthesis	F_4
4G162-5	new-type *B. juncea*	3G42-2	Hybrid Synthesis	F_4
4G164-4	new-type *B. juncea*	3G47-1	Hybrid Synthesis	F_4
5G06	new-type *B. juncea*	4G03-1	Hybrid Synthesis	F_5
5G09	new-type *B. juncea*	4G159-1	Hybrid Synthesis	F_5
5G10	new-type *B. juncea*	4G159-2	Hybrid Synthesis	F_5
5G11	new-type *B. juncea*	4G160-1	Hybrid Synthesis	F_5
5G13	new-type *B. juncea*	4G164-4	Hybrid Synthesis	F_5
5G16	new-type *B. juncea*	4G158-1	Hybrid Synthesis	F_5
5G17	new-type *B. juncea*	4G161-5	Hybrid Synthesis	F_5
5G18	new-type *B. juncea*	4G162-1	Hybrid Synthesis	F_5
5G19	new-type *B. juncea*	4G162-5	Hybrid Synthesis	F_5

"-" represent the unknown

Table S2 The molecular markers linked to the clubroot resistance gene used in this study

Name	Primer sequence（5'-3'）	CR-Genes	Source
CR-m090a	F: GCAAAGATCGGCGAAGAAGA<br>R: TGCAGACACATTCGAACAAACA	*PbBa8.1*	Chen et al. 2013
CR-BSA3	F: GAGTGACATCGAAAATCAGATAGC<br>R: CCTAAATGGAAAGGCTTGGC	*PbBa1.1*	Chen et al. 2013
CR-S14R14	F: CACGACGTTGTAAAACGACTTAAATCCGGACGTGAAAT<br>R: AAGAGGAAGAAGCTCCTGA	*BraA.CR.b*	Hirani et al. 2018
CR-S17R17	F: CACGACGTTGTAAAACGACCATCCACTTTGGACTGTGA<br>R: TCAGAGAACTCAGCTCGTG	*BraA.CR.b*	Hirani et al. 2018

Table S3 Results of molecular marker genotyping for the F_2-F_5

F_2	F_3	F_4	F_5	CR-BSA3	CR-S14R14	CR-m090a	CR-S17R17
2G53				7/42	4/42	11/42	21/42
	3G49-1			3/15	6/15	6/15	11/15
		4G158-1		5/10	7/10	8/10	7/10
			5G16	2/10	3/10	6/10	5/10
		4G158-2		0/10	0/10	0/10	1/10
		4G158-3		1/10	2/10	3/10	2/10
		4G158-4		5/10	3/10	3/10	5/10
		4G158-5		2/10	2/10	3/10	4/10
	3G49-2			4/22	4/22	14/22	7/22
		4G159-1		5/10	8/10	8/10	2/10
			5G09	0/10	6/10	7/10	7/10
		4G159-2		1/10	2/10	8/10	4/10
			5G10	0/10	6/10	8/10	9/10
		4G159-3		1/10	2/10	4/10	5/10
	3G49-3			1/15	5/15	2/15	2/15
		4G160-1		0/10	8/10	8/10	8/10
			5G11	5/10	9/10	10/10	7/10
	3G49-4			4/10	5/10	8/10	4/10
		4G04-1		1/10	6/10	4/10	7/10
2G55				7/33	7/33	10/33	17/33
	3G47-1			2/10	2/10	6/10	8/10
		4G164-4		3/10	3/10	8/10	8/10
			5G13	6/10	6/10	8/10	10/10
	3G47-2			5/10	3/10	7/10	9/10
		4G07-1		0/10	0/10	8/10	9/10
	3G47-3			5/10	3/10	8/10	9/10
		4G05-1		1/10	10/10	7/10	4/10
	3G47-4			2/10	8/10	8/10	7/10
		4G03-1		0/10	5/10	7/10	4/10
			5G06	3/10	10/10	10/10	8/10
	3G47-5			1/10	0/10	8/10	9/10
		4G14-1		4/10	0/10	6/10	4/10
	3G47-6			2/10	8/10	8/10	7/10
		4G16-6		1/10	4/10	7/10	0/10
	3G47-7			3/10	2/10	5/10	8/10
		4G15-1		6/10	3/10	0/10	7/10
2G54				7/41	22/41	13/41	19/41
	3G42-1			3/18	1/18	4/18	4/18
		4G161-5		2/10	2/10	8/10	8/10
			5G17	0/10	3/10	3/10	4/10
	3G42-2			2/9	2/9	6/9	1/9

F_2	F_3	F_4	F_5	CR-BSA3	CR-S14R14	CR-m090a	CR-S17
		4G162-5		2/10	3/10	5/10	6/10
			5G19	7/10	1/10	6/10	7/10
		4G162-1		1/10	2/10	5/10	5/10
			5G18	6/10	0/10	4/10	4/10
	3G42-3			7/10	5/10	8/10	2/10
		4G01-1		0/10	1/10	6/10	3/10
	3G42-4			2/10	4/10	8/10	4/10
		4G02-1		0/10	4/10	6/10	3/10
	3G42-5			1/10	7/10	8/10	8/10
	3G42-6			8/10	8/10	7/10	8/10
		4G12-1		1/10	4/10	3/10	4/10
